# Supplementary material for: Stunting Status of Ever-Married Adolescent Mothers and Its Association with Childhood Stunting with a Comparison by Geographical Region in Bangladesh
Source: Int J Environ Res Public Health. 2022 May 31;19(11):6748. doi: 10.3390/ijerph19116748 (PMC9180893; doi:10.3390/ijerph19116748)
Supplement: Supplementary file 1 [file ijerph-19-06748-s001.zip › ijerph-1702776-supplementary.pdf]

Supplementary Table S1. General characteristics of participants stratified by administrative region in Bangladesh (2007-2018).

Indicators, % (n)	Administrative region in Bangladesh						
	Barisal	Chittagong	Dhaka[!]	Khulna	Rajshahi[$]	Sylhet	Overall
**Household characteristics**							
Place of residence							
Urban	26.65	30.42	40.16	32.01	26.68	27.08	30.88
Rural	73.35	69.58	59.84	67.99	73.32	72.92	69.12
Wealth index							
Poorest	23.29	10.63	15.56	12.46	24.63	21.58	18.22
Poorer	29.58	15.78	18.22	19.26	26.12	25.89	22.19
Middle	22.97	25.10	16.89	27.20	21.80	19.49	22.06
Richer	16.68	26.08	25.20	25.40	17.94	18.01	21.72
Richest	7.48	22.40	24.14	15.68	9.51	15.03	15.81
Number of HH members[1]	5.95	6.72	5.55	5.56	5.47	7.10	5.92
Improved toilet	43.36	54.28	50.58	51.80	49.54	42.47	49.40
Source of drinking water	79.48	78.64	82.24	74.12	82.01	78.21	79.69
Religion							
Others	4.98	5.56	3.46	9.35	9.77	12.20	7.33
Muslim	95.02	94.44	96.54	90.65	90.23	87.80	92.67
**Ever married adolescent girl characteristics**							
Height in cm[1]	151.13	151.43	150.58	151.83	150.82	150.05	150.99
Weight in kg[1]	46.35	47.26	46.31	47.14	45.86	44.22	46.28
Age in year[1]	17.95	18.16	18.05	17.95	17.86	18.21	18.01
Height-for-age z score[1]	1.78	1.75	1.86	1.67	1.82	1.96	1.80
BMI-for-age z score[1]	0.44	0.34	0.41	0.33	0.48	0.69	0.44
Stunting	38.73	37.98	43.16	33.56	40.02	47.27	39.89
Maternal education							
No education	4.01	6.62	7.11	2.74	4.68	13.99	5.99
Primary	26.00	23.22	30.59	16.71	23.19	40.48	25.72
Secondary	57.42	60.67	53.99	70.63	63.75	41.37	59.38
Higher	12.57	9.48	8.31	9.92	8.38	4.17	8.91
Husband's education							
No education	10.83	14.23	16.09	12.65	16.30	27.68	15.74
Primary	36.62	28.05	35.37	30.41	34.09	42.26	33.88
Secondary	37.38	44.81	37.83	41.27	36.81	24.55	37.95
Higher	15.17	12.92	10.70	15.68	12.80	5.51	12.44
Husband's age[1]	26.37	26.85	25.91	25.85	25.31	27.75	26.13
Had the ability to take decisions herself (or jointly with her husband)							
i. Own health care	41.52	48.08	50.68	46.69	51.02	40.15	47.64
ii. Major household purchases	33.37	34.17	43.51	37.13	43.29	29.24	38.38
iii. Visits to her family or relatives	37.33	39.00	47.20	40.84	46.84	36.52	42.59
All of the three decisions	21.70	25.33	30.81	24.85	30.29	22.58	26.99
Attitudes to domestic violence	66.41	71.95	75.27	67.04	71.67	71.13	71.08
At least 4 ANC visits from medically trained provider	9.75	12.10	12.50	16.43	15.01	9.23	13.02
Use of contraceptive method	53.41	35.65	47.94	54.77	53.37	29.61	47.32
Delivery type							

Caesarean section	14.48	15.23	23.60	24.39	16.26	8.54	17.60
Non-caesarean	85.52	84.77	76.40	75.61	83.74	91.46	82.40
**Child's characteristics**							
Child's age in months[1]	17.77	16.21	17.75	18.97	19.70	14.14	17.84
Child's sex							
Male	48.37	48.88	54.92	50.76	50.94	55.56	51.47
Female	51.63	51.12	45.08	49.24	49.06	44.44	48.53
Childhood stunting	39.18	31.75	35.79	32.35	36.79	40.73	35.78

[!]Mymensing division was added with Dhaka,
[$]Rangpur division was added with Rajshahi,
[1]Mean
